# Design of Inkjet-Printed RFID-Based Sensor on Paper: Single- and Dual-Tag Sensor Topologies

**DOI:** 10.3390/s18061958

**Published:** 2018-06-17

**Authors:** Sangkil Kim, Apostolos Georgiadis, Manos M. Tentzeris

**Affiliations:** 1 Department of Electronics Engineering, Pusan National University, Busan, 46241, Korea; 2 Heriot Watt University, Edinburgh Campus, Edinburgh EH14 4AS, UK; apostolos.georgiadis@ieee.org; 3 Department of Electrical and Computer Engineering, Georgia Institute of Technology, 777 Atlantic Dr. NW, Atlanta, GA 30332, USA; etentze@ece.gatech.edu

**Keywords:** inkjet-printed sensor, RFID-enabled sensor, wireless sensor, paper electronics, self-sustainable wireless sensor

## Abstract

The detailed design considerations for the printed RFID-based sensor system is presented in this paper. Starting from material selection and metallization method, this paper discusses types of RFID-based sensors (single- & dual-tag sensor topologies), design procedures, and performance evaluation methods for the wireless sensor system. The electrical properties of the paper substrates (cellulose-based and synthetic papers) and the silver nano-particle-based conductive film are thoroughly characterized for RF applications up to 8 GHz. The reported technology could potentially set the foundation for truly “green”, low-cost, scalable wireless topologies for autonomous Internet-of-Things (IoT), bio-monitoring, and “smart skin” applications.

## 1. Introduction

Paper has found an almost ubiquitous use in numerous applications due to its low-cost, fabrication maturity, and availability in various forms customized to specific needs and conditions. The cost of paper is typically 20~150 times lower than other polymer-based materials like polyethylene terephtalate (PET) and polyimide (PI), with comparable flexibility to such polymers [1]. Paper is also an environmentally friendly (“green”) material, because it is recyclable and contains no phenolic compounds. Over the last decade, paper substrates have received growing attention as electronic substrates due to the ever-increasing demand for low cost, flexible, and green technologies [1]. The advantages of paper for electronics can be further enhanced in light of large-area printed electronics fabrication techniques, such as inkjet printing, as demonstrated in many reported research efforts [2]. Inkjet printing technology is an additive process which does not produce by-products, such as strong acids (wet etching) or chips (milling machines), while it can realize resolutions of below 25 µm [3,4]. Silver nano-particle ink is widely utilized for printing conductive traces for paper electronics, although many types of nano-particle inks are made from copper (Cu) [5] and gold (Au) [6], due to their low sintering temperatures and high conductivities. These attractive properties of inkjet-printed paper electronics (ease of low cost fabrication and prototyping) make the technology a strong candidate for the easy-to-scale implementation of next generation electronics, such as the Internet of things (IoT), radio frequency identification (RFID)-based sensor technology, and printed passive/active electronics [7].

RFID-based sensing systems have also attracted significant interest as printing technology evolves. They have relatively simple sensor system architecture, and the RFID tag is highly compatible with printing technology. Some commercial RFID antennas are already printed on polymer substrates (e.g., PET, polyimide, etc.) for the mass production of RFID antennas at low cost [8,9]. In addition, most RFID tags operate in passive mode by enabling an energy harvesting feature which is a built-in function of the commercial passive RFID chips. It does not need complicated electronic circuitry or power sources like a battery to maintain the deployed RFID tags. The RFID chip harvests radiated RF energy from the RFID reader through the antenna to activate the chip. Therefore, a self-sustainable, standalone wireless sensor system having a longer life and lower maintenance costs can be realized by converging printing, passive RFID, and sensor technologies. It is also a very practical approach since it does not need any specific equipment or system to realize the RFID-based sensor system. It needs only one feature at the RFID reader side of sweeping the Tx frequency. It requires only 6.5% of frequency tunability at UHF RFID frequency band (880~940 MHz), which is not a challenging feature. The collected data can be easily analyzed and processed by a signal processor at the base band to detect an event. Therefore, the RFID-based sensor system presented in this paper can be integrated with prebuilt RFID system without any significant system change or incompatibility.

The novelty of this paper is the presentation of the design procedure and analysis method of the RFID-based printed sensors. This paper has thoroughly analyzed operation principles of the printed RFID-based sensors, and provided parametric qualitative evaluation technique of the RFID-based sensors using normalized read range analysis. Electrical properties of paper substrates popular for RFID tag printing are also thoroughly characterized over the broad frequency band up to 8 GHz. Cellulose-based and synthetic Teslin papers are characterized, and their electrical properties, such as dielectric constant (*ε_r_*) and loss tangent (tan *δ*), are presented. The inkjet printing technology optimized for the silver nano-particle film printing was chosen as a fabrication method of RFID tags in order to take advantage of the scalable and low-cost properties of printing technology [10]. Design considerations for the two sensor topologies (single- and dual-tag sensors) are discussed in detail throughout the case study of the reported research efforts [11,12]. The theory of read range estimation method as a quantitative method for evaluating the performance of the RFID-based sensor system is also presented, with detailed design examples. 

## 2. Paper Substrates and Inkjet-Printed Conductive Film

There are numerous types of papers (e.g., cellulose-based, synthetic Teslin, etc.). It is critical to select a paper substrate that is compatible or can be functionalized [13] to accommodate all stages of the fabrication process (e.g., good adhesion for the inkjet-printed materials, withstand the sintering process without cracking, excessive shrinkage/warpage and performance deterioration) in order to print electronics on paper operating at microwave frequencies. Another important factor is the accurate characterization of the electrical properties of the printed material over a broad frequency, temperature, and humidity range. Without loss of generality, the electrical properties of two commonly-used, paper-based substrates are presented as a reference for typical RF designs: cellulose-based photo paper and synthetic Teslin.

### 2.1. Cellulose-Based Photo-Paper

For the paper fabrication process, the cellulose-base fibers or pulps are fed to a machine, and a paper web is formed. The moisture in the paper web is mechanically removed by pressing and drying utilizing air or heat. Additives, such as chalk or china clay, are added for better quality of printing or writing on paper. However, the cellulose-based paper without any surface functionalization or coating is not a good choice for direct nanoparticle-based ink printing, although it has many advantages like process maturity, simplicity, and its recyclable nature. Printed nano-particle ink penetrates the paper substrate resulting in a discontinuous (disconnected) conductive layer.

**Figure 1:**
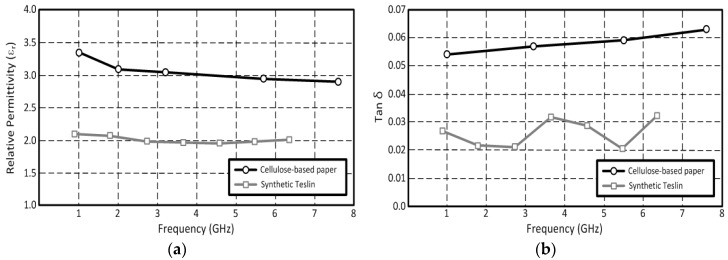
(**a**) Relative permittivity (*ε_r_*) of cellulose-based paper (black) and synthetic paper (gray) and (**b**) loss tangent (tan *δ*) of cellulose-based paper (black) and synthetic Teslin (gray).

However, the paper has a lot of advantages as a substrate for passive/active electronics, such as antennas, RFIDs, and sensors. The paper is a flexible, renewable, and bio-compatible material, and it is able to withstand harsh environments (such as the humidity) when it is treated with proper chemicals [14]. The electrical properties of a commonly used 254 µm thick cellulose-based Kodak photo-paper have been thoroughly studied in [4]; those are shown in Figure 1 at frequency band of 1~8 GHz. The paper is characterized by both T-resonator and ring resonator methods [15,16]. The relative permittivity (*ε_r_*) of the paper is about 2.9~3.4 and the loss tangent (tan δ) is about 5.3 × 10^−2^~6.2 × 10^−2^ at 1~8 GHz. The relative permittivity of the paper (*ε_r_* = 3.4) is similar to that of other commonly used RF substrates, such as LCP (*ε_r_* = 2.9, tan *δ* = 2.5 × 10^−3^), polyimide (*ε_r_* = 3.5, tan *δ* = 2.6 × 10^−3^), and FR4 (*ε_r_* = 4.4, tan *δ* = 1.8 × 10^−2^) at 1 GHz while the tan *δ* of the cellulose-based paper (tan *δ* = 5.3 × 10^−2^) is significantly higher than those materials due to its fiber-based organic composition. However, such a high value of the tan *δ* is not an important parameter for certain designs like low Q-factor designs, because the electromagnetic fields are not tightly bounded/confined within the glossy substrate [17]. In this case, the conductivity (σ), thickness and roughness of the inkjet-printed metallic structures are the critical/limiting factors for the realization of high-performance RF electronics on paper.

### 2.2. Synthesized Polymeric Paper: Teslin

The cellulose-based paper substrate suffers from durability issue during long-term exposure to harsh environment or high temperature, despite its advantages as a substrate material for printed electronics. In this case, synthetic papers, such as Teslin which is commonly used in the production of ID and security cards, could be good alternatives. Teslin features the most advantages of the various cellulose-based papers, because it is a flexible, environmentally friendly, and recyclable material [18]. Plus, it is able to stand much higher sintering temperatures (up to 220 °C) compared to the cellulose-based paper. Teslin has been originally synthesized for printing applications, and is a petroleum-free material, thus being non-toxic and recyclable. Teslin was characterized by utilizing a printed ring resonator technique; the ε_r_ and the tan *δ* of a 254 μm thick Teslin substrate are shown in Figure 1 (gray lines). The relative permittivity of Teslin is about 2.0, and the loss tangent is about 2.2 × 10^−2^ at 1~8 GHz. It should be noted that the Teslin has lower ε_r_ and *δ* values compared to those of cellulose-based paper.

### 2.3. Inkjet-Printed Conductive Film Using Silver Nano-Particles

Silver-nanoparticle inks are widely used for inkjet-printed electronics on various substrates, including paper, due to their inherent capability of relatively low-temperature sintering temperature and their relatively higher conductivity values compared to other nano-particle-based inks such as Copper (Cu) or Gold (Au) inks [3,4]. The properties of a commonly.used silver nano-particle ink have been thoroughly studied and reported in [4]. The sintering process, silver nano-particle ink concentration, and the number of printed layers are the main factors affecting the conductivity of the inkjet-printed conductive film. The sintering process not only burns off the impurities/polymer coating around the nano-particles, but also increases the bond of printed nano-particles with the substrate. Although thermal sintering is widely used due to its simplicity and low-cost, various other types of sintering processes, such as laser, UV flash lamp and microwave sintering [19,20], have demonstrated uniform performance and high conductivity values for a relatively short duration of sintering time. Typically, higher nano-particle concentrations and number of printed layers increase the particle density of the printed traces, resulting in higher conductivity values. However, high nano-particle concentration of the ink easily clogs the printer nozzles. Therefore, it is necessary to take a thorough printing experimentation before large-scale/-area implementation. In this section, silver nano-particle ink was printed on glass substrate in order to minimize effect of surface roughness. This paper discusses the electrical properties of the inkjet-printed silver nano-particles; mechanical performance was reported in [21]. 

**Figure 2:**
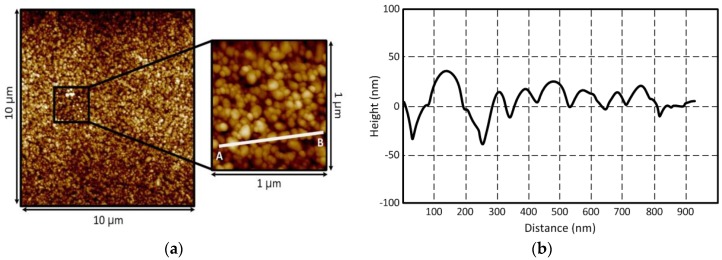
Surface of the inkjet-printed silver nano-particles: (**a**) AFM images of 10 μm × 10 μm and 1 μm × 1 μm areas, and (**b**) cross section of the line $\bar{\mathrm{AB}}$.

Figure 2 shows an atomic force microscope (AFM) scanned image of an inkjet-printed conductive silver nano-particle film. The sample was printed using a 10 pL ink cartridge with a 20 µm (1024 dpi) droplet spacing on a glass, and the sample was thermally sintered at 150 °C for 2 h in a convection oven at atmospheric pressure. For printing, Dimatix DMP2800 printer and 1 pL and 10 pL cartridges was utilized, and it was kept at a distance of 800 μm from the surface of the substrate on a printing plate of the printer. The printer-head angle was adjusted to 4.2° in order to achieve a print resolution of 1270 dpi. Cabot conductive ink CCI-300 was jetted at the temperature of 36 °C, while the glass substrate was maintained at 50 °C. The silver nano-particle ink consisted of ethanol and ethylene glycol (ethanediol), concentration of silver nano-particle was 20 wt %. Size of the silver nano-particles was less than 150 nm, and viscosity was 10~13 cP.

**Figure 3:**
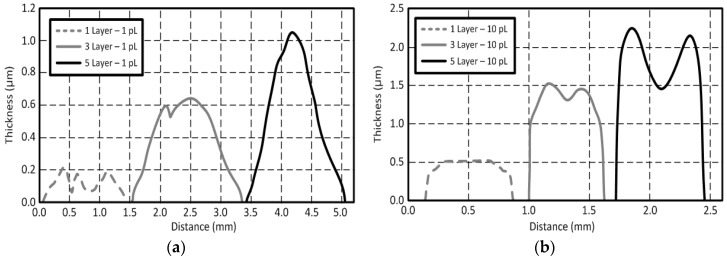
An inkjet-printed silver nano-particle conductive film as a function of the number of printed layers and of the ink droplet volume: (**a**) 1 pL (width: 1.0 mm), (**b**) 10 pL (width: 0.5 mm).

**Figure 4:**
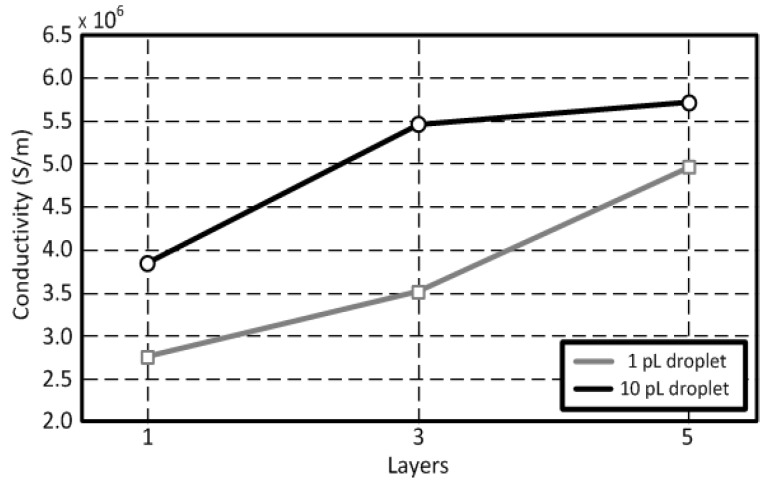
Conductivity values (σ_Ag_, 150 °C) of an inkjet-printed silver-nanoparticle trace after the thermal sintering at 150 °C with a 20 µm drop spacing (1024 dpi).

The measured root mean squared (*R_q_*) and arithmetic average (*R_a_*) roughness values are 14.4 nm and 11.4 nm, respectively. Profiles of the inkjet-printed traces depending on a volume of the ink droplet (1 pL & 10 pL) for the same printer setting and sintering process are shown in Figure 3. A Dekktak profilometer is utilized to measure the thickness of the traces. Each printed layer adds about 200 nm of thickness for 1 pL cartridge (Figure 3a) and 500 nm of thickness for 10 pL cartridge (Figure 3b). In Figure 3b, the coffee ring effect was observed due to the excessive solvent (printed by the 10 pL cartridge), while the effect was not shown in Figure 3a (printed by 1pL cartridge) [22,23]. The thickness of the printed traces on the glass ranges from 0.2~3.0 µm. The conductivity values (σ) of the printed traces can be extracted through $\sigma =l\cdot {\left(A\cdot R\right)}^{-1}$ with the respective values shown in Figure 3, where *A* is the cross-section area, *l* is the length of the printed trace and *R* is the measured resistance between two points of the trace. The extracted DC conductivity values are around 4.96 × 10^6^ S/m for 1 pL cartridge and 5.70 × 10^6^ S/m for 10 pL cartridge when the thermal sintering temperature is 150 °C. The conductivity value of the inkjet-printed silver-nanoparticles can be increased to 1.2 × 10^7^ S/m when the sintering temperature is 200 °C. This corresponds to 19.05% of a bulk silver’s conductivity value (σ_Ag_ = 6.3 × 10^7^ S/m). The conductivity value of the trace for 10 pL cartridge is higher than that for 1 pL cartridge. This is because the 10 pL cartridge can print a much thicker layer than 1 pL cartridge, since the volume of printed ink from the 10 pL cartridge is much larger than that of a 1 pL cartridge. The extracted conductivity values of the printed silver are shown in Figure 4. The surface resistance (*R_s_*) at 900 MHz can be extracted from the measurement shown in Figure 4 using $R_{s}=\sqrt{\pi f\mu /\sigma }$ [24]. *R_s_* of the printed silver nano-particle layer is about 25.17 mΩ, while that of the bulk silver is about 7.96 mΩ. The printed silver nano-particle film can be considered as a good conductor at UHF RFID frequency band, since σ≫ωε, although it has relatively lower *R_s_*.

## 3. Design of Printed RFID-Based Sensor

### 3.1. Operation Principle of a RFID-Based Sensor and Its Topology: Single and Dual Tag Sensors

**Figure 5:**
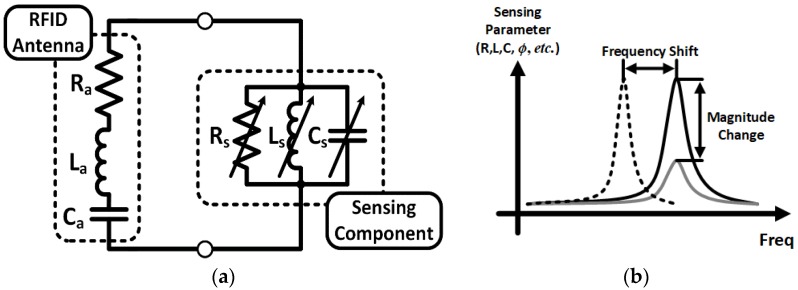
(**a**) Equivalent circuit model of a RFID-based sensor tag and (**b**) frequency response of sensing component.

**Figure 6:**
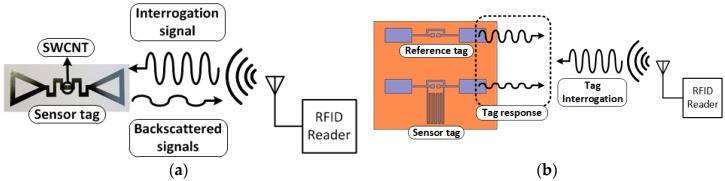
RFID-based sensor topology: (**a**) Single-tag SWCNT gas sensor and (**b**) dual-tag capacitive haptic sensor.

The RFID-based sensor basically takes advantage of impedance change (phase or magnitude) of the sensing component (antenna load), as shown in Figure 5. A simplified equivalent circuit model of an RFID-based sensor tag is shown in Figure 5a. An equivalent circuit model of a dipole antenna (a series RLC resonant circuit) was chosen, since most of RFID systems use dipole antennas as their radiator. Any electrical changes of the sensing component are directly reflected to the RFID antenna because the sensing component is a load of the antenna. There are three main methods to detect events through the loading effect of the sensing component to the RFID antenna: detecting (1) magnitude of the load impedance, (2) frequency shift, and (3) phase shift (Figure 5b). The detecting frequency shift method utilizes multiple resonators or needs broad band frequency sweep to detect resonant frequency. It requires a complicated interrogator system and broadband matching to manage the frequency shift. This topology is not compatible with RFID chip-enabled topology, which operates at RFID frequencies at 868 or 915 MHz. It is unable to detect the sensors due to the mismatch between the antenna and the chip when the sensor component shifts the resonant frequency of the antenna too far away from the operating frequency (868 MHz or 915 MHz). The detecting phase shift method is much more challenging for the RFID-based sensor system, since it requires high quality of calibration. Therefore, the impedance magnitude variation is widely utilized RFID-based sensor topology, due to its ease of realization and event detection. In the case of chip-less RFID-based single-tag sensor, the variation of electrical properties such as R, L, C at RFID frequency (868 MHz or 915 MHz) is a critical design factor for decision making. The magnitude of impedance change depending on the variation of sensing target (e.g., gas, chemical, protein etc.) is directly reflected to the sensitivity of the sensor system, since most of the chip-less RFID-based sensors utilize back-scattering communication, as shown in Figure 6a. Therefore, it is important to track the ratio of the incident power to reflected power. It is intuitive that a bigger impedance variation results in the more sensitivity of the wireless sensor.

The RFID-based sensor system can be implemented by single or dual tag topologies. Both sensing topologies utilize the same operation principle of detecting electrical property variation of a sensing component. One of the biggest advantages of the single tag RFID sensor tag is that it is relatively easy to build a sensor system because of its simplicity. A sensing component can be directly printed onto the antenna (input port or radiator) or integrated in the RFID chip. However, usually it requires high quality of calibration at the interrogator (or the reader), because the sensor tag is exposed to the surrounding environment without protection or shielding. Thorough wireless/electromagnetic characterization of ambient environment where the sensor tags are deployed is important to prevent false alarms or malfunctioning of the single tag RFID-based sensor system. 

Different from the single tag operation, the dual tag RFID-based sensor consists of two RFID tags for the stable operation of the sensor system. In general, the dual tag RFID-based sensor consists of a reference and a sensor tag, as shown in Figure 6b. The reader is able to distinguish the tag responses when the RFID tags have RFID chip. The reference tag returns the same response (the same magnitude of the reflected power) before/after the event, but the sensor tag returns different power levels because of the loading effect due to the sensing component. Therefore, events can be detected by comparing the responses from the tags without thorough calibration or wireless characterization of ambient environment, because both tags are exposed to the same surrounding environment effect. However, the dual tag sensing topology has a larger tag size than the single sensing one, because it has two tags on the same plane. Crosstalk or coupling between the two tags is also a critical parameter for sensor tag miniaturization.

### 3.2. Fully Printed Chip-Less Single-Tag SWCNT NH_3_ Gas Sensor

**Figure 7:**
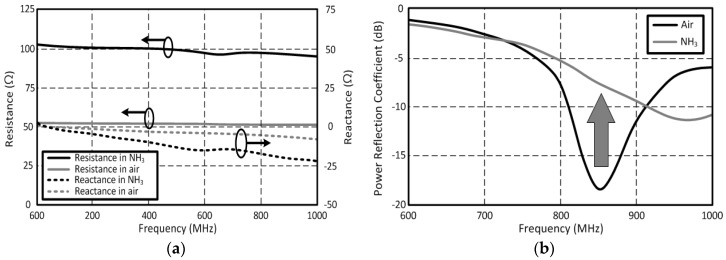
(**a**) Measured impedances of SWCNT film which printed 25 layers (thickness: 7 μm) in air/NH_3_ and (**b**) the power reflection coefficient of the RFID-based sensor with the SWCNT film before/after the NH_3_ gas exposure.

An inkjet-printed RFID-based chip-less carbon nanotube (CNT) gas sensor is presented as a design example and a proof of concept for a single-tag RFID-based sensor (Figure 6a) [11]. For the ink formulation, CNT powder was dispersed in DI water to make a concentration of 5 mg/mL. The formulated CNT ink was sonicated in order to form a uniform dispersion in the ink. Glycerol was added to meet the viscosity (10~13 cP) requirements of the printer (DMP2800). For the purpose of homogenization, the formulated CNT ink was sonicated before printing. The sensor tag is interrogated by an RFID interrogator (reader), and the backscattered power is monitored by the reader. The sensor tag has a sensing component which consists of an inkjet-printed single wall carbon nanotube (SWCNT) film acting like a tunable resistor and a parallel capacitor [25]. The resistance value changes as the concentration of the target gas such as ammonia (NH_3_) varies. The event decision (or gas detection) can be made by monitoring the backscattered power level as a result of the variations in the electrical characteristics of the loaded SWCNT film. The sensor tag was placed in an air-controlled glass chamber and coaxial lines are connected to VNA (outside the chamber) to measure the impedance change of the sensor. The sensor was exposed to 4% NH_3_, 96% N_2_ gas for the measurement, and complex impedance values were measured over the frequency range from 600 MHz to 1 GHz. The measured impedance of the printed SWCNT film on photo paper is shown in Figure 7a. The thickness of the 25 times printed SWCNT film is about 7 µm. At 868 MHz which is located within the European RFID standard frequency band, the impedance value of the inkjet-printed SWCNT film was 51.6 − *j*6.1 Ω in air (no presence of ammonia, NH_3_), while the impedance value changed to 97 − *j*18.8 Ω when the SWCNT sensor was exposed to NH_3_. The variation of the reflected power (a ratio of the incident power to the reflected power) from the RFID-enabled SWCNT gas sensor is shown in Figure 7b to demonstrate the operation of the single tag RFID-enabled sensor. The reflected power level of the sensor tag in air was −18.4 dB, while that of the sensor tag in NH_3_ was −7.6 dB, due to the mismatch between the SWCNT film and the RFID antenna.

### 3.3. Dual-Tag RFID-Based Capacitive Haptic Sensor

**Figure 8:**
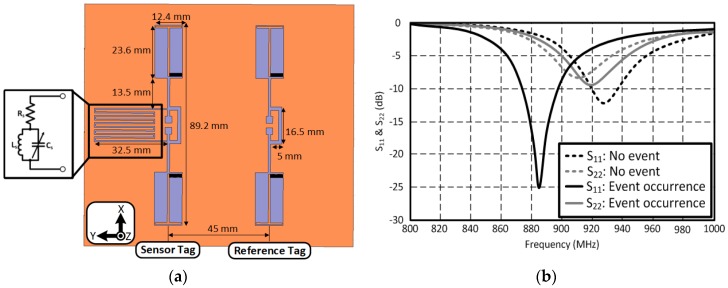
(**a**) Dual-tag sensor design and (**b**) frequency response of each tag.

An inkjet-printed dual-tag RFID-based sensor is also shown as a design example. The presented dual-tag sensor utilizes the impedance variation of the sensing component to detect an event [12]. It consists of a sensor and a reference tag; the two RFID chips (NXP’s SL3ICS1002/1202) mounted at the input port of each dipole antenna resonate at 915 MHz. The sensor tag is integrated with a capacitive haptic sensor which can be modeled as a series resistance and a parallel LC network (Figure 8a). The self-resonant frequency of the sensing component is designed at the same resonant frequency of the antenna before the event detection (in the air). The input impedance of the sensing component is high enough to be considered an open circuit at the self-resonant frequency when it is seen from the antenna. The frequency response of the two tags with/without the sensing component (sensing/reference tags) is very close, as shown in Figure 8b (dashed lines). In Figure 8b, antennas are conjugate matched to the RFID chip impedance (13.3 − *j*122 Ω) at UHF RFID band. For the simulation, a full wave 3D simulator, Ansys HFSS v11.1, was utilized for this analysis. Port 1 is an excitation port (location of RFID chip) of a sensor tag, and port 2 is that of a reference tag. Therefore, S_11_ represents the reflection coefficients between an RFID chip and the sensor antenna, while S_22_ means reflection coefficients between an RFID chip and the reference tag. However, the difference between the frequency responses of the tags is clearly noticeable when an event occurs. The resonant frequency of the sensor tag shifted to lower frequency due to the loading effect of the sensing component. This is because that the self-resonant frequency of the sensing component is not located at the operation frequency of the RFID antenna (not a high impedance value at the operation frequency of the RFID antenna). 

### 3.4. Performance Analysis Parameter: Read Range Evaluation

One of the most popular evaluation parameters of the RFID-based sensor system is a read range, because it includes a concept of the required minimum power to active the RFID. The read range of a single RFID tag can be estimated from the Friis equation based on the antenna gain, the input impedance of the antenna, and the RFID chip [10]. It is a function of the complex antenna impedance (*Z_a_ = R_a_ + jX_a_* Ω), the RFID antenna gain (*G_r_*) the complex RFID chip impedance (*Z_c_ = R_c_ + jX_c_* Ω), and the power transmission coefficient (*τ = 4R_a_R_c_/|Z_c_ + Z_a_|*^2^). For a single RFID tag, a normalized read range can be expressed, as shown in (1) [26]. *R*_0_ is the estimated range of a tag, which has a gain of 0 dBi, and which is matched to the RFID chip at the operation frequency.

(1) $$\frac{R}{R_{0}}=\sqrt{G_{r}\tau }$$

The above equation can be extended to 2-port system (*n* = 1, 2) to analyze the dual tag RFID-based sensor, as shown in (2) and (3).

(2) $$\frac{R_{n}}{R_{0}}=\sqrt{G_{n}\tau_{n}}$$

(3) $$\tau_{n}=4R_{c}R_{A,nn}\frac{{\left|{\left[Y\right]}_{n}g\right|}^{2}}{{\left|g_{n}\right|}^{2}}$$

where *τ_n_* is the power transmission coefficient at port *n* of the multiport system which determines the quality of matching between the antennas and the load (a sensing component or a RFID chip). The **[*Y*]*_n_*** term denotes the *n*-th row of the admittance matrix (***Y***), of the antenna system; ***g*** is a column vector of the normalized antenna gains ($g_{n}$ is a *n*-th element of ***g*** matrix), and *R_A,nn_* is a real part of the antenna input impedance at port *n* [27]. 

**Figure 9:**
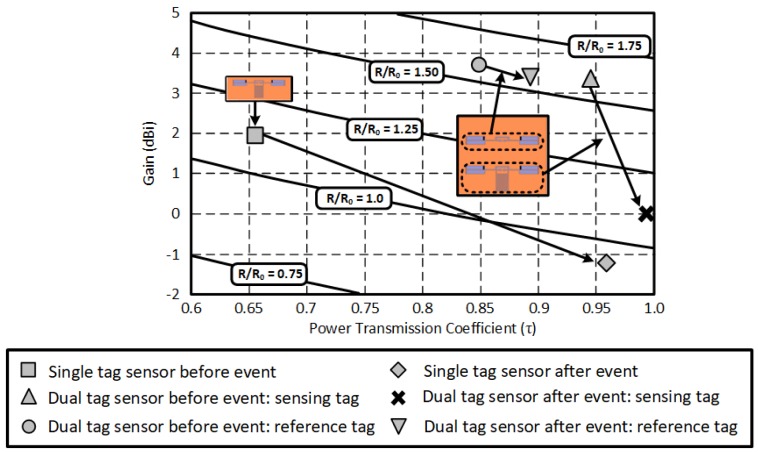
Read range analysis of the RFID-based single and dual tag sensors.

The discussed theoretical analysis based on the estimated read range of the RFID-enabled sensors is applied to the presented RFID-based sensor systems, as shown in Figure 9. The direction of the arrows indicates the occurrence of an event (before → after). The read range performance of the sensing tags (dual- and single tag sensors) was significantly affected by an event, while the reference tag of the dual tag sensor maintains its performance. The largest read range variation was observed from a single tag sensor. This means that it is more sensitive than the dual tag sensor topology, although it easily interacts with all the ambient environment. For the dual tag sensing topology, it is clear that the read range difference between the reference and the sensor tag is notable. The read range of the reference tag does not change after the event, but that of the sensor tag drops significantly. The event decision can be easily made by comparing the response of the two tags. There are three major possibilities when a reader interrogates the dual-tag RFID-enabled sensor: (1) reader detects both tags, (2) reader detects only one tag, and (3) reader detects no tags. The proposed dual-tag RFID-based sensor needs to detect both tags to compare their frequency responses. In the case of other two, read range of the RFID system should be improved. It can be improved by increasing Tx power or antenna gain at the reader side. For the Rx side (reference and sensor tags), battery-assisted active RFID tag, or more directive antenna such as a microstrip patch antenna, are able to increase the read range of the proposed dual-tag sensor system.

## 4. Conclusions

This paper has introduced the materials and fabrication method for the inkjet-printed RFID-based sensor system, including paper substrates (cellulose and synthetic papers) and conductive nano-particle film. The concept, design procedure, and design examples of self-sustainable standalone RFID-based wireless sensors are discussed in detail. The theory of the read range estimation method for multi RFID-based sensor tags is also presented with mathematical calculation and analysis examples. The RFID-based sensor system discussed in this work can be scalable to any other wireless energy harvesting-enabled self-sustainable sensor system operating at any other frequency bands without loss of generality. This technology could potentially set the foundation for truly “green”, low-cost, scalable wireless topologies for autonomous Internet-of-Things (IoT), bio-monitoring and “smart skin” applications.
